# (*E*)-3,3′-(Diazene-1,2-di­yl)bis­(1-methyl-1,4,5,6-tetra­hydro­pyrrolo­[3,4-*c*]pyrazol-5-ium) dinitrate dihydrate

**DOI:** 10.1107/S1600536811053347

**Published:** 2011-12-17

**Authors:** Jin-Mei Chen, Hong Zhao

**Affiliations:** a School of Chemistry and Chemical Engineering, Southeast University, Nanjing 210096, People’s Republic of China

## Abstract

The title compound, C_12_H_18_N_8_^2+^·2NO_3_^−^·2H_2_O, was synthesized unexpectedly from 3-amino-1-methyl-1,4,5,6-tetra­hydro­pyrrolo­[3,4-*c*]pyrazol-5-ium chloride and cerium(IV) ammonium nitrate. The cation has a crystallographically imposed centre of symmetry. In the crystal, the ions and water mol­ecules are linked *via* O—H⋯N, N—H⋯O and O—H⋯O hydrogen bonds into a three-dimensional network.

## Related literature

For background to potential anti­cancer kinase inhibitors, see: Fancelli *et al.* (2005 ▶); Gadekar *et al.* (1968 ▶). For a related structure, see: Xia *et al.* (2011 ▶).
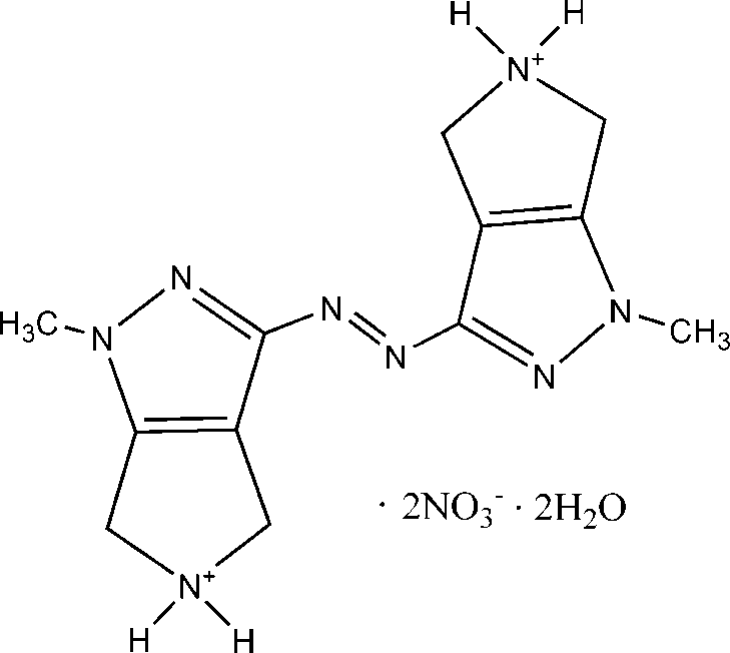


## Experimental

### 

#### Crystal data

C_12_H_18_N_8_^2+^·2NO_3_^−^·2H_2_O
*M* *_r_* = 434.40
Triclinic, 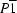

*a* = 6.2344 (12) Å
*b* = 7.7725 (16) Å
*c* = 9.7071 (19) Å
α = 99.56 (3)°
β = 92.49 (3)°
γ = 92.84 (3)°
*V* = 462.64 (16) Å^3^
*Z* = 1
Mo *K*α radiation
μ = 0.13 mm^−1^
*T* = 295 K
0.22 × 0.16 × 0.12 mm

#### Data collection

Rigaku SCXmini diffractometer
4322 measured reflections
1811 independent reflections
1479 reflections with *I* > 2σ(*I*)
*R*_int_ = 0.034

#### Refinement

*R*[*F* ^2^ > 2σ(*F* ^2^)] = 0.048
*wR*(*F* ^2^) = 0.131
*S* = 1.08
1811 reflections
145 parameters
H atoms treated by a mixture of independent and constrained refinement
Δρ_max_ = 0.28 e Å^−3^
Δρ_min_ = −0.21 e Å^−3^

### 

Data collection: *CrystalClear* (Rigaku, 2005 ▶); cell refinement: *CrystalClear*; data reduction: *CrystalClear*; program(s) used to solve structure: *SHELXS97* (Sheldrick, 2008 ▶); program(s) used to refine structure: *SHELXL97* (Sheldrick, 2008 ▶); molecular graphics: *SHELXTL/PC* (Sheldrick, 2008 ▶); software used to prepare material for publication: *SHELXTL/PC*.

## Supplementary Material

Crystal structure: contains datablock(s) I, global. DOI: 10.1107/S1600536811053347/rz2680sup1.cif

Structure factors: contains datablock(s) I. DOI: 10.1107/S1600536811053347/rz2680Isup2.hkl

Supplementary material file. DOI: 10.1107/S1600536811053347/rz2680Isup3.cml

Additional supplementary materials:  crystallographic information; 3D view; checkCIF report

## Figures and Tables

**Table 1:** Hydrogen-bond geometry (Å, °)

*D*—H⋯*A*	*D*—H	H⋯*A*	*D*⋯*A*	*D*—H⋯*A*
O1—H1*E*⋯N4^i^	0.95 (3)	1.98 (3)	2.895 (2)	163 (2)
N2—H2*B*⋯O1^ii^	0.90	1.94	2.802 (3)	159
N2—H2*A*⋯O1^iii^	0.90	2.44	2.970 (2)	118
N2—H2*A*⋯O3^iv^	0.90	2.18	2.894 (3)	136
O1—H1*F*⋯O2	0.85 (4)	1.97 (4)	2.819 (2)	173 (3)

Symmetry codes: (i) 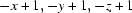; (ii) 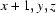; (iii) 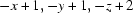; (iv) 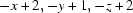.

**Fig. 1.**
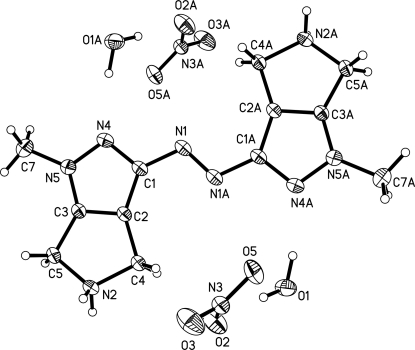
The molecular structure of the title compound showing displacement ellipsoids drawn at the 30% probability level. Atoms with suffix A are generated by the symmetry operation (1-x, 1-y, 1-z).

**Fig. 2.**
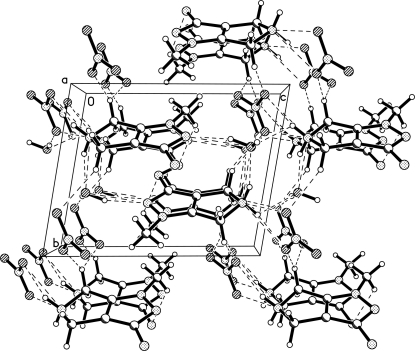
Packing diagram of the title compound, showing the structure along the a axis. Dashed lines indicate hydrogen bonds.

## supplementary crystallographic information

### Comment

Tetrahydropyrrolo[3,4-*c*]pyrazol derivatives are used as anticancer kinase inhibitors (Xia *et al.*, 2011; Fancelli *et al.*, 2005; Gadekar *et al.*, 1968). The title compound was synthesized unexpectedly from 3-amino-1-methyl-1,4,5,6-tetrahydropyrrolo[3,4-*c*]pyrazol-5-ium chloride and cerium(IV) ammonium nitrate, and its crystal structure is presented herein.

The molecular structure of the title compound is shown in Fig. 1. The cation lies on a crystallographic inversion centre located at the centre of the diazene fragment. The dihedral angle between the fused pyrrole and pyrazole rings is 4.46 (12)°. In the crystal structure, the ions and water molecules are linked *via* O—H···N, N—H···O and O—H···O hydrogen bonds into a three-dimensional network. (Table 1; Fig. 2).

### Experimental

3-Amino-1-methyl-1,4,5,6-tetrahydropyrrolo[3,4-*c*]pyrazol-5-ium chloride (0.35 g, 2 mmol) and cerium(IV) ammonium nitrate (0.28 g, 0.5 mmol) were dissolved in 95% ethanol (25 ml). The solution was filtered and left at room temperature for 10 days. Yellow prism crystals suitable for X-ray analysis were obtained by slow evaporation of the solvent.

### Refinement

The water H atoms were located in a difference Fourier map and refined freely. All other H atoms were placed in calculated positions and refined using a riding model approximation, with C—H=0.96–0.92 Å,N—H=0.97 Å, and with *U*_iso_(H) = 1.2*U*_eq_(C, N) or 1.5*U*_eq_(C) for methyl groups.

### Crystal data

**Table:**

C_12_H_18_N_8_^2+^·2NO_3_^−^·2H_2_O	*Z* = 1
*M**_r_* = 434.40	*F*(000) = 228
Triclinic, *P*1	*D*_x_ = 1.559 Mg m^−^^3^
Hall symbol: -P 1	Mo *K*α radiation, λ = 0.71073 Å
*a* = 6.2344 (12) Å	Cell parameters from 4309 reflections
*b* = 7.7725 (16) Å	θ = 3.1–27.2°
*c* = 9.7071 (19) Å	µ = 0.13 mm^−^^1^
α = 99.56 (3)°	*T* = 295 K
β = 92.49 (3)°	Prism, yellow
γ = 92.84 (3)°	0.22 × 0.16 × 0.12 mm
*V* = 462.64 (16) Å^3^	

### Data collection

**Table:**

Rigaku SCXmini diffractometer	1479 reflections with *I* > 2σ(*I*)
Radiation source: fine-focus sealed tube	*R*_int_ = 0.034
graphite	θ_max_ = 26.0°, θ_min_ = 3.1°
Detector resolution: 13.6612 pixels mm^-1^	*h* = −7→7
CCD_Profile_fitting scans	*k* = −9→9
4322 measured reflections	*l* = −11→11
1811 independent reflections	

### Refinement

**Table:**

Refinement on *F*^2^	Primary atom site location: structure-invariant direct methods
Least-squares matrix: full	Secondary atom site location: difference Fourier map
*R*[*F*^2^ > 2σ(*F*^2^)] = 0.048	Hydrogen site location: inferred from neighbouring sites
*wR*(*F*^2^) = 0.131	H atoms treated by a mixture of independent and constrained refinement
*S* = 1.08	*w* = 1/[σ^2^(*F*_o_^2^) + (0.060*P*)^2^ + 0.1401*P*] where *P* = (*F*_o_^2^ + 2*F*_c_^2^)/3
1811 reflections	(Δ/σ)_max_ < 0.001
145 parameters	Δρ_max_ = 0.28 e Å^−^^3^
0 restraints	Δρ_min_ = −0.21 e Å^−^^3^

### Special details

**Table:**

Geometry. All e.s.d.'s (except the e.s.d. in the dihedral angle between two l.s. planes) are estimated using the full covariance matrix. The cell e.s.d.'s are taken into account individually in the estimation of e.s.d.'s in distances, angles and torsion angles; correlations between e.s.d.'s in cell parameters are only used when they are defined by crystal symmetry. An approximate (isotropic) treatment of cell e.s.d.'s is used for estimating e.s.d.'s involving l.s. planes.
Refinement. Refinement of *F*^2^ against ALL reflections. The weighted *R*-factor *wR* and goodness of fit *S* are based on *F*^2^, conventional *R*-factors *R* are based on *F*, with *F* set to zero for negative *F*^2^. The threshold expression of *F*^2^ > σ(*F*^2^) is used only for calculating *R*-factors(gt) *etc*. and is not relevant to the choice of reflections for refinement. *R*-factors based on *F*^2^ are statistically about twice as large as those based on *F*, and *R*- factors based on ALL data will be even larger.

### Fractional atomic coordinates and isotropic or equivalent isotropic displacement parameters (Å^2^)

**Table:**

	*x*	*y*	*z*	*U*_iso_*/*U*_eq_	
C1	0.7436 (3)	0.6228 (2)	0.52005 (18)	0.0324 (4)	
C2	0.8295 (3)	0.6536 (2)	0.65780 (17)	0.0301 (4)	
C3	1.0187 (3)	0.7441 (2)	0.65097 (18)	0.0308 (4)	
C4	0.7930 (3)	0.6264 (3)	0.80235 (18)	0.0370 (5)	
H4A	0.7592	0.5043	0.8065	0.044*	
H4B	0.6790	0.6959	0.8426	0.044*	
C5	1.1457 (3)	0.7872 (3)	0.78510 (19)	0.0388 (5)	
H5A	1.1556	0.9119	0.8199	0.047*	
H5B	1.2891	0.7446	0.7779	0.047*	
C7	1.2218 (3)	0.8565 (3)	0.4629 (2)	0.0467 (5)	
H7A	1.3142	0.9190	0.5383	0.070*	
H7B	1.1674	0.9371	0.4068	0.070*	
H7C	1.3016	0.7717	0.4062	0.070*	
N1	0.5569 (2)	0.5305 (2)	0.45680 (15)	0.0353 (4)	
N2	1.0105 (3)	0.6894 (2)	0.87401 (16)	0.0404 (4)	
H2A	0.9912	0.7595	0.9556	0.049*	
H2B	1.0803	0.5968	0.8932	0.049*	
N3	0.6426 (3)	0.1158 (3)	0.86872 (18)	0.0456 (5)	
N4	0.8749 (3)	0.6936 (2)	0.43628 (16)	0.0363 (4)	
N5	1.0436 (2)	0.7685 (2)	0.51941 (15)	0.0340 (4)	
O1	0.1944 (3)	0.3662 (2)	0.86511 (17)	0.0527 (4)	
O2	0.6074 (3)	0.2635 (2)	0.93400 (19)	0.0651 (5)	
O3	0.8117 (3)	0.0492 (3)	0.89667 (19)	0.0733 (6)	
O5	0.5152 (3)	0.0407 (3)	0.77657 (19)	0.0761 (6)	
H1E	0.167 (4)	0.323 (3)	0.769 (3)	0.068 (8)*	
H1F	0.323 (6)	0.344 (4)	0.886 (3)	0.084 (10)*	

### Atomic displacement parameters (Å^2^)

**Table:**

	*U*^11^	*U*^22^	*U*^33^	*U*^12^	*U*^13^	*U*^23^
C1	0.0333 (9)	0.0308 (9)	0.0313 (9)	0.0052 (8)	−0.0060 (7)	0.0007 (7)
C2	0.0293 (9)	0.0284 (9)	0.0310 (9)	0.0027 (7)	−0.0034 (7)	0.0012 (7)
C3	0.0301 (9)	0.0286 (9)	0.0325 (9)	0.0031 (7)	−0.0016 (7)	0.0024 (7)
C4	0.0318 (9)	0.0437 (11)	0.0341 (10)	−0.0039 (8)	−0.0051 (8)	0.0065 (8)
C5	0.0311 (9)	0.0456 (11)	0.0379 (10)	−0.0057 (8)	−0.0047 (8)	0.0061 (8)
C7	0.0461 (12)	0.0463 (12)	0.0498 (12)	0.0040 (10)	0.0094 (10)	0.0122 (9)
N1	0.0359 (9)	0.0330 (8)	0.0347 (8)	0.0038 (7)	−0.0089 (6)	0.0009 (6)
N2	0.0365 (9)	0.0528 (10)	0.0302 (8)	−0.0027 (8)	−0.0063 (7)	0.0052 (7)
N3	0.0430 (10)	0.0540 (11)	0.0378 (9)	−0.0108 (9)	−0.0043 (8)	0.0078 (8)
N4	0.0403 (9)	0.0373 (9)	0.0301 (8)	0.0048 (7)	−0.0042 (7)	0.0034 (6)
N5	0.0353 (8)	0.0332 (8)	0.0336 (8)	0.0042 (7)	0.0007 (6)	0.0051 (6)
O1	0.0513 (10)	0.0694 (11)	0.0375 (8)	0.0156 (8)	0.0006 (7)	0.0056 (7)
O2	0.0619 (11)	0.0573 (11)	0.0706 (11)	0.0123 (9)	−0.0215 (8)	−0.0018 (9)
O3	0.0658 (11)	0.0726 (12)	0.0727 (12)	0.0239 (10)	−0.0173 (9)	−0.0139 (9)
O5	0.0587 (11)	0.0946 (14)	0.0639 (11)	−0.0315 (10)	−0.0129 (9)	−0.0043 (10)

### Geometric parameters (Å, °)

**Table:**

C1—N4	1.340 (3)	C7—N5	1.451 (3)
C1—C2	1.397 (2)	C7—H7A	0.9600
C1—N1	1.398 (2)	C7—H7B	0.9600
C2—C3	1.353 (3)	C7—H7C	0.9600
C2—C4	1.479 (2)	N1—N1^i^	1.258 (3)
C3—N5	1.336 (2)	N2—H2A	0.9000
C3—C5	1.474 (2)	N2—H2B	0.9000
C4—N2	1.517 (2)	N3—O5	1.221 (2)
C4—H4A	0.9700	N3—O3	1.237 (2)
C4—H4B	0.9700	N3—O2	1.250 (2)
C5—N2	1.500 (3)	N4—N5	1.344 (2)
C5—H5A	0.9700	O1—H1E	0.95 (3)
C5—H5B	0.9700	O1—H1F	0.85 (4)
			
N4—C1—C2	110.64 (16)	N5—C7—H7A	109.5
N4—C1—N1	116.89 (16)	N5—C7—H7B	109.5
C2—C1—N1	132.44 (17)	H7A—C7—H7B	109.5
C3—C2—C1	104.04 (16)	N5—C7—H7C	109.5
C3—C2—C4	111.36 (15)	H7A—C7—H7C	109.5
C1—C2—C4	144.60 (17)	H7B—C7—H7C	109.5
N5—C3—C2	109.44 (16)	N1^i^—N1—C1	112.49 (18)
N5—C3—C5	136.16 (17)	C5—N2—C4	111.95 (14)
C2—C3—C5	114.38 (16)	C5—N2—H2A	109.2
C2—C4—N2	100.74 (14)	C4—N2—H2A	109.2
C2—C4—H4A	111.6	C5—N2—H2B	109.2
N2—C4—H4A	111.6	C4—N2—H2B	109.2
C2—C4—H4B	111.6	H2A—N2—H2B	107.9
N2—C4—H4B	111.6	O5—N3—O3	120.6 (2)
H4A—C4—H4B	109.4	O5—N3—O2	120.7 (2)
C3—C5—N2	99.89 (14)	O3—N3—O2	118.73 (18)
C3—C5—H5A	111.8	C1—N4—N5	105.67 (14)
N2—C5—H5A	111.8	C3—N5—N4	110.19 (15)
C3—C5—H5B	111.8	C3—N5—C7	128.79 (17)
N2—C5—H5B	111.8	N4—N5—C7	120.99 (16)
H5A—C5—H5B	109.5	H1E—O1—H1F	107 (3)
			
N4—C1—C2—C3	−0.7 (2)	N4—C1—N1—N1^i^	−179.69 (18)
N1—C1—C2—C3	176.94 (18)	C2—C1—N1—N1^i^	2.8 (3)
N4—C1—C2—C4	179.0 (2)	C3—C5—N2—C4	−11.9 (2)
N1—C1—C2—C4	−3.4 (4)	C2—C4—N2—C5	13.2 (2)
C1—C2—C3—N5	0.9 (2)	C2—C1—N4—N5	0.20 (19)
C4—C2—C3—N5	−178.88 (15)	N1—C1—N4—N5	−177.85 (14)
C1—C2—C3—C5	−177.86 (15)	C2—C3—N5—N4	−0.9 (2)
C4—C2—C3—C5	2.3 (2)	C5—C3—N5—N4	177.5 (2)
C3—C2—C4—N2	−9.2 (2)	C2—C3—N5—C7	−179.34 (17)
C1—C2—C4—N2	171.1 (2)	C5—C3—N5—C7	−0.9 (3)
N5—C3—C5—N2	−172.5 (2)	C1—N4—N5—C3	0.39 (19)
C2—C3—C5—N2	5.9 (2)	C1—N4—N5—C7	179.01 (16)

Symmetry codes: (i) −*x*+1, −*y*+1, −*z*+1.

### Hydrogen-bond geometry (Å, °)

**Table:**

*D*—H···*A*	*D*—H	H···*A*	*D*···*A*	*D*—H···*A*
O1—H1E···N4^i^	0.95 (3)	1.98 (3)	2.895 (2)	163 (2)
N2—H2B···O1^ii^	0.90	1.94	2.802 (3)	159.
N2—H2A···O1^iii^	0.90	2.44	2.970 (2)	118.
N2—H2A···O3^iv^	0.90	2.18	2.894 (3)	136.
O1—H1F···O2	0.85 (4)	1.97 (4)	2.819 (2)	173 (3)

Symmetry codes: (i) −*x*+1, −*y*+1, −*z*+1; (ii) *x*+1, *y*, *z*; (iii) −*x*+1, −*y*+1, −*z*+2; (iv) −*x*+2, −*y*+1, −*z*+2.
